# Successful Surgical Management of Giant Cardiomegaly in a Patient With Multivalvular Heart Disease: A Case Report and Literature Review

**DOI:** 10.7759/cureus.88352

**Published:** 2025-07-20

**Authors:** Tao-An Chen, Ya-Ting Chuang, Hua-Yu Lin, Ching-Chou Pai

**Affiliations:** 1 Chest Medicine, Show Chwan Memorial Hospital, Changhua, TWN; 2 Critical Care, Show Chwan Memorial Hospital, Changhua, TWN; 3 Cardiovascular Surgery, Show Chwan Memorial Hospital, Changhua, TWN

**Keywords:** aortic stenosis, cardiomegaly, cardiothoracic ratio (ctr), mechanical circulatory support, valve replacement surgery

## Abstract

Cardiomegaly, particularly in its extreme form, is a rare but clinically significant manifestation of chronic valvular heart disease. While a cardiothoracic ratio (CTR) greater than 0.50 indicates cardiac enlargement, a CTR exceeding 0.80 is exceptionally uncommon and typically associated with advanced disease. We report the case of a 70-year-old man with severe aortic and mitral stenosis who developed acute decompensated heart failure (HF) and exhibited profound cardiomegaly (CTR ≥ 0.80). We initiated intervention upon the patient's presentation to the emergency department and closely followed his clinical progression through surgery and subsequent recovery until discharge from the ward. The patient required mechanical circulatory support, including an intra-aortic balloon pump (IABP) and extracorporeal membrane oxygenation (ECMO), followed by successful aortic and mitral valve replacement, as well as tricuspid annuloplasty. Despite postoperative hemodynamic instability requiring delayed sternal closure, the patient was ultimately weaned off support and discharged in stable condition. This case underscores the prognostic value of CTR as a non-invasive marker of cardiac remodeling and highlights the potential for recovery in even extreme anatomical presentations when timely and aggressive interventions are undertaken.

## Introduction

Cardiomegaly, historically referred to as 'cor bovinum,' is a descriptive term most commonly used in forensic pathology, though it has also appeared in clinical contexts [1]. However, the term "cor bovinum" is now infrequently used in clinical practice. The term, dating back to the 18th century, is associated with severe cardiac hypertrophy resulting from chronic pressure overload conditions such as aortic stenosis (AS) [1,2]. Aortic stenosis is the most prevalent valvular heart disease in developed countries and often leads to progressive left ventricular (LV) hypertrophy as a compensatory response to increased afterload [3]. This concentric remodeling results in decreased LV compliance and diastolic dysfunction, eventually progressing to heart failure (HF) if left untreated. Even after aortic valve replacement (AVR), persistent LV dysfunction is associated with a poor prognosis [3].

The association between AS and 'cor bovinum' was first described in 1942 [2]. In modern practice, extreme cardiomegaly continues to indicate advanced valvular heart disease with significant prognostic implications. We present the case of a 70-year-old man with severe AS and mitral stenosis (MS) who developed profound cardiomegaly (cardiothoracic ratio (CTR) ≥ 0.80) and acute decompensated HF, requiring advanced mechanical circulatory support and complex valve surgery. This case underscores the clinical significance of cardiomegaly as both a radiological and physiological manifestation of chronic valvular pathology.

## Case presentation

A 70-year-old man with a past medical history of hypertension, gout, heart disease with severe AS and MS post percutaneous balloon mitral valvotomy, and complete right bundle branch block (RBBB) with a permanent pacemaker presented with acute-onset dyspnea. He denied allergies, recent travel, or exposure to infectious contacts. He was a non-smoker and did not consume alcohol or betel nut.

**Figure 1:**
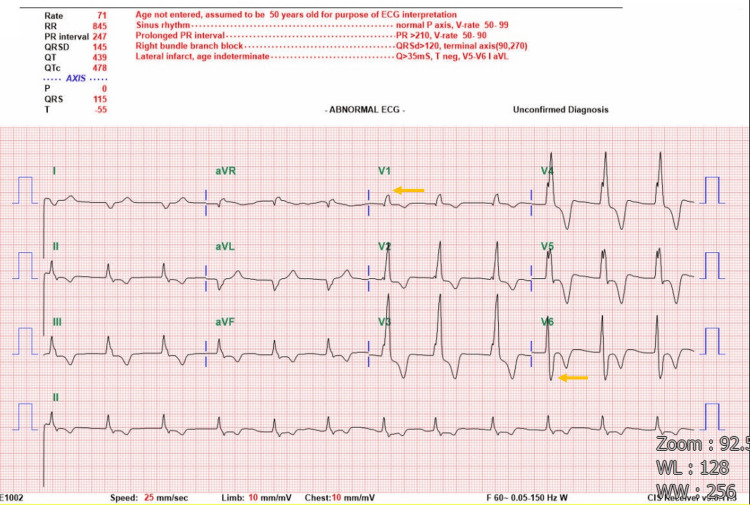
The ECG shows sinus rhythm, prolonged PR interval, right axis deviation, and complete RBBB (orange arrow indicates the area of abnormal heart rhythm).

At the referring hospital, he was intubated for respiratory failure. Arterial blood gas analysis revealed hypoxemia and hypercapnia (pH 7.277, PaCO₂ 62.6 mmHg, PaO₂ 47.4 mmHg, O₂ saturation 73.7%). Laboratory evaluation showed elevated blood urea nitrogen (33 mg/dL), creatinine (1.21 mg/dL), N terminal pro B type natriuretic peptide (1092 pg/mL), troponin T (26.8 ng/L), and C reactive protein (3.8 mg/dL), with a hemoglobin of 12.5 g/dL (Table 1). The SARS-CoV-2 and influenza antigen testing were negative. Electrocardiography (ECG) showed sinus rhythm, prolonged PR interval, right axis deviation, and complete RBBB (Figure 1). The detailed ECG parameters are summarized in Table 2.

**Table 1:** Laboratory results at the time of admission

Test	Result (on admission)	Reference range
Blood urea nitrogen	33 mg/dL	6-20 mg/dL
Creatinine	1.21 mg/dL	0.7-1.2 mg/dL
Creatine phospho kinase	58 U/L	39-308 U/L
Sodium	139 mmol/L	136-145 mmol/L
Potassium	4.0 mmol/L	3.5-5.1 mmol/L
N terminal pro B type natriuretic peptide	1092 pg/mL	<125 pg/mL
Creatine kinase MB	2.17 ng/mL	<6.22 ng/mL
Troponin T	26.8 ng/L	<14 ng/L
Albumin	4.1 gm/dL	3.5-5.2 gm/dL
Lactic acid	11 mg/dL	4.5-19.8 mg/dL
C reactive protein	3.8 mg/dL	<0.5 mg/dL
Procalcitonin	0.1 ng/mL	<0.5 ng/mL
Hemoglobin	12.5 g/dL	14.0-18.0 g/dL

**Table 2:** Summary of ECG parameters and automated interpretation

Parameter	Value	Remarks
Heart rate (bpm)	71	Normal resting heart rate
RR Interval (ms)	845	Consistent with sinus rhythm
PR interval (ms)	247	Prolonged PR interval (>200 ms)
QRS duration (ms)	145	Prolonged; consistent with RBBB
Corrected QT (QTc, ms)	439	Within the upper normal limit
P axis (°)	0	Normal atrial depolarization axis
QRS axis (°)	115	Rightward axis
T axis (°)	–55	Inverted T wave axis, potentially ischemic
Interpretation	RBBB, lateral infarct	Based on automated analysis; requires clinical correlation

**Figure 2:**
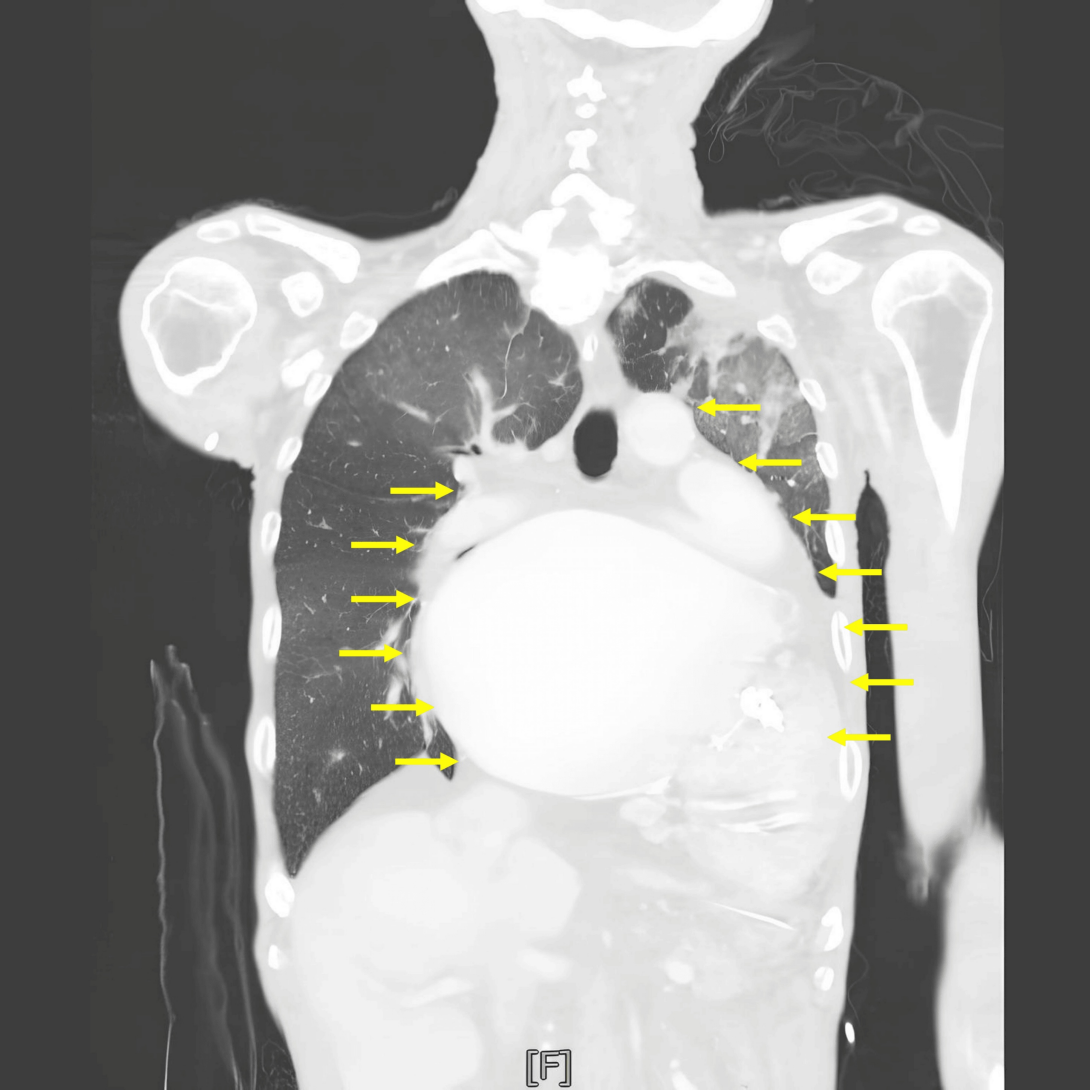
Computed tomography angiography shows severe left atrial enlargement, bilateral pleural effusions, left lung consolidation, and right lung ground-glass opacities (yellow arrows indicate the border of the heart)

**Figure 3:**
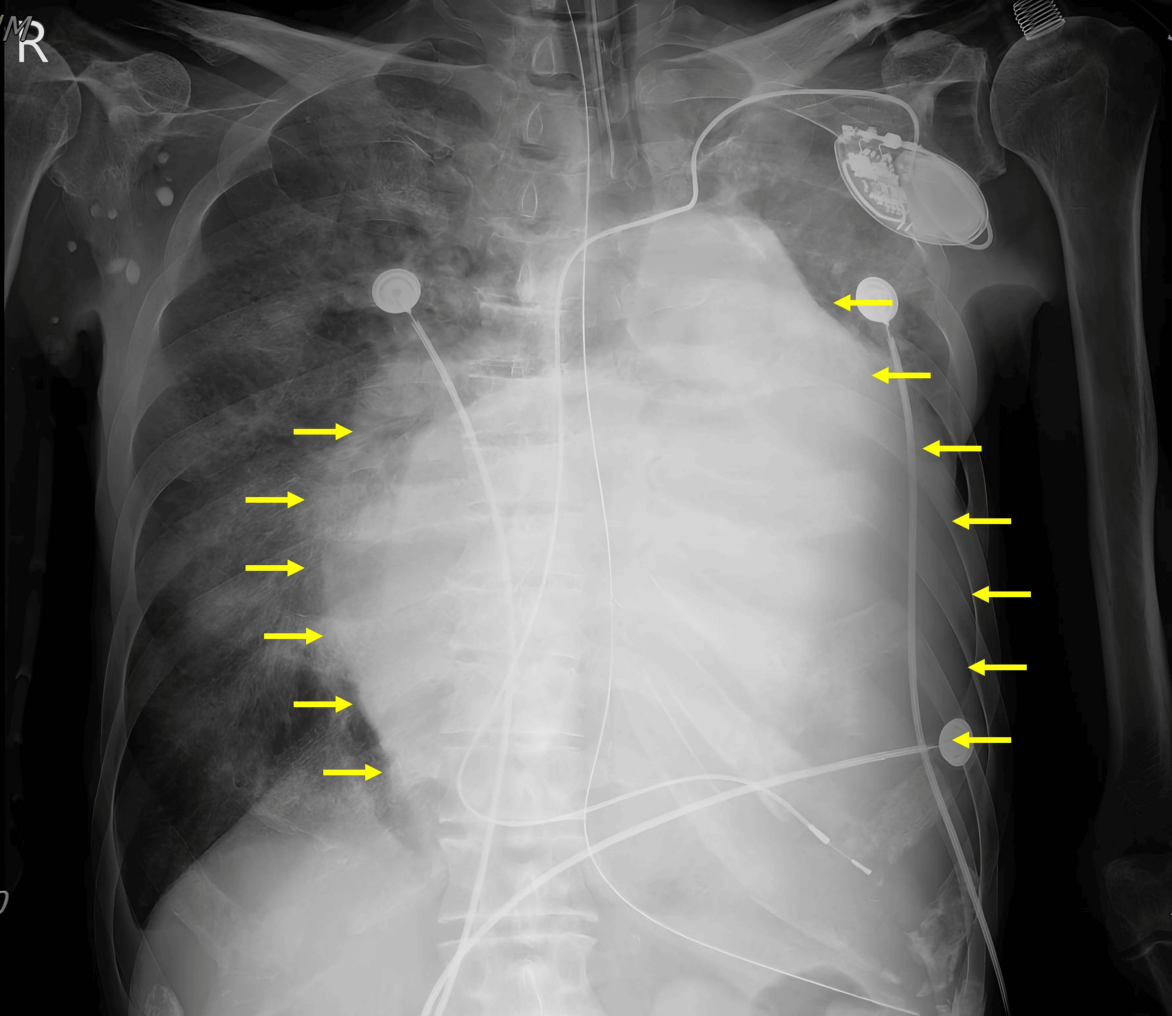
Chest X-ray reveals marked cardiomegaly, bilateral perihilar haziness, obscured left basal and retrocardiac regions, and signs of emphysema (yellow arrow indicates the border of the heart)

Computed tomography angiography demonstrated severe left atrial enlargement, bilateral pleural effusions, left lung consolidation, and right lung ground-glass opacity (Figure 2). Concurrent chest imaging also revealed cardiomegaly, with a CTR of at least 0.80 (Figure 3). The patient was emergently placed on an intra-aortic balloon pump (IABP) with an ECG trigger set at 1:1. He was then transferred to the intensive care unit under the care of the cardiovascular surgery team.

Preoperative color Doppler echocardiography revealed severe AS (aortic valve area (AVA): 0.71 cm²; peak gradient: 65.6 mmHg; peak velocity (Vmax): 4.05 m/s) and severe mitral stenosis (mitral valve area (MVA): 0.54 cm²; peak gradient: 32 mmHg) (Table 3). Following hemodynamic stabilization, the patient underwent aortic and mitral valve replacement with mechanical prostheses (Sorin M 20 mm and 33 mm, respectively) and tricuspid valve annuloplasty under general anesthesia on hospital day four. The procedure necessitated high-dose heparinization, vasopressor and inotropic support, diuretics, and transfusion of multiple blood products, including fresh frozen plasma, platelet apheresis, and packed red blood cells. Multiple central venous lines and chest drains were placed. Due to persistent hemodynamic instability, the chest was temporarily left open postoperatively. The patient was transferred back to the intensive care unit with continued support via extracorporeal membrane oxygenation (ECMO), IABP, and inotropic agents.

**Table 3:** Pre and postoperative comparison of color Doppler echocardiography

Parameter	Preoperative	Postoperative	Reference range
Aortic valve Vmax (AV_Vmax)	4.05 m/s	3.01 m/s	<1.3 m/s
Aortic valve pressure gradient (AV_PG)	65.6 mmHg	36.4 mmHg	< 20 mmHg
Aortic valve area (AVA)	0.71 cm²	N/A	>1.5 cm²
Aortic regurgitation severity	Moderate	Mild	-
Mitral valve Vmax (MV_Vmax)	2.83 m/s	2.26 m/s	<1.7 m/s
Mitral valve pressure Gradient (MV_PG)	32.0 mmHg	20.5 mmHg	< 5 mmHg
Mitral valve area (MVA)	0.54 cm²	N/A	>1.5 cm²
Mitral Regurgitation Severity	Mild	Trivial	-

The patient’s condition gradually improved. On hospital day seven, he was successfully weaned from ECMO support, allowing for subsequent sternal closure. After several weeks of intensive care management, the patient's condition stabilized, and he was discharged home with good clinical status on hospital day 25.

## Discussion

Our patient presented with an exceptionally elevated CTR ≥ 0.8 on posteroanterior chest radiography, well above the conventional threshold for severe cardiomegaly, which is defined as CTR > 0.60 [4]. Notably, a previously reported case described a cardiothoracic ratio approaching 0.90, attributed to severe left atrial enlargement and structural cardiac distortion [5]. Although the patient in that case underwent valve replacement surgery, the outcome was unfavorable, and the patient ultimately did not survive [5]. The CTR is calculated as the ratio of the maximal horizontal cardiac diameter to the maximal internal thoracic diameter on a posteroanterior chest X-ray [4]. It is commonly classified as normal (0.42-0.50), mildly enlarged (0.50-0.55), moderately enlarged (0.55-0.60), and severely enlarged (>0.60) [4].

Extreme cardiomegaly, particularly from left-sided chamber dilation, represents a compensatory response to chronic hemodynamic overload but carries substantial prognostic implications. The timing of valve replacement in patients with valvular heart disease is critical, as delays in surgical intervention may lead to irreversible remodeling and fatal outcomes [5]. An epidemiological study has shown that LV hypertrophy, identified by ECG or echocardiography, is a dose-dependent and independent predictor of sudden cardiac death [6]. Similarly, cardiomegaly has been identified as a strong prognostic indicator for all-cause and cardiovascular mortality in patients undergoing hemodialysis [7].

Among available noninvasive tools, CTR remains a widely used surrogate for detecting LV hypertrophy [4,8]. It correlates significantly with various demographic and biochemical parameters, and higher CTR has been associated with increased risks of mortality, cardiovascular events, and composite outcomes [8]. Recently, artificial intelligence (AI) tools have shown promise in automating CTR calculation with accuracy comparable to human readers, facilitating faster and consistent radiographic screening in emergency and critical care settings [9,10]. The AI models have demonstrated high agreement with human observers in segmenting cardiac and pulmonary structures and calculating CTR values [9,10]. When validated under clinical conditions, these tools show great potential as rapid screening or decision-support systems, particularly in time-critical environments such as emergency departments and intensive care units, where immediate expert interpretation may be unavailable [9].

Although cardiomegaly is generally defined radiographically as a CTR > 0.50, a CTR ≥ 0.80 is rare and clinically significant. Notably, our patient presented in a state of hemodynamic collapse, yet prompt initiation of IABP support allowed for successful surgical intervention. Owing to profound intraoperative instability and the need for ongoing circulatory support, delayed sternal closure was required. Reports describing valve surgery in patients with a CTR ≥ 0.80 are extremely limited, and few have documented favorable postoperative outcomes in such settings. This case adds to the limited body of literature describing successful surgical treatment of giant cardiomegaly involving multivalvular disease, underscoring the potential for recovery through timely and aggressive management, even in anatomically extreme cases.

## Conclusions

Extreme cardiomegaly, as defined by a CTR ≥ 0.80, is rarely reported and often portends a poor prognosis in patients with advanced valvular heart disease. The CTR remains a valuable, noninvasive screening and prognostic tool in this setting. This case demonstrates that with early recognition, prompt circulatory support, and definitive surgical intervention, favorable outcomes are achievable even in anatomically severe presentations. Moreover, the successful management of this patient reinforces the importance of individualized perioperative strategies, including delayed sternal closure and multimodal hemodynamic support, in achieving recovery from giant cardiomegaly.
